# Elucidation of the Molecular Basis and Cellular Functions of Vinculin-Actin Directional Catch Bonding

**DOI:** 10.21203/rs.3.rs-2334490/v1

**Published:** 2023-01-12

**Authors:** Venkat R. Chirasani, Mohammad Ashhar I. Khan, Juilee N. Malavade, Nikolay V. Dokholyan, Brenton D. Hoffman, Sharon L. Campbell

**Affiliations:** 1 Department of Biochemistry & Biophysics, University of North Carolina at Chapel Hill, Chapel Hill, NC, USA.; 2 Department of Biomedical Engineering, Duke University, Durham, NC, USA.; 3 Department of Pharmacology, Department of Biochemistry & Molecular Biology, Department of Chemistry, Penn State College of Medicine, Hershey, PA, USA.; 4 Department of Cell Biology, Duke University, Durham, NC, USA; 5 Lineberger Comprehensive Cancer Center, University of North Carolina at Chapel Hill, Chapel Hill, NC, USA.

## Abstract

The ability of cells and tissues to differentially resist or adapt to mechanical forces applied in distinct directions is mediated by the ability of load-bearing proteins to preferentially maintain physical linkages in certain directions. However, the molecular basis and biological consequences of directional force-sensitive binding are unclear. Vinculin (Vcn) is a load-bearing linker protein that exhibits directional catch bonding due to interactions between the Vcn tail domain (Vt) and filamentous (F)-actin. We developed a computational approach to predict Vcn residues involved in directional catch bonding and produced a set of associated Vcn variants with unaltered Vt structure, actin binding, or phospholipid interactions. Incorporation of these variants into Vcn biosensors did not perturb Vcn conformation, but reduced Vcn loading consistent with loss of directional catch bonding. Expression of Vcn variants perturbed the coalignment of FAs and F-actin and directed cell migration, establishing key cellular functions for Vcn directional catch bonding.

## Teaser

This work provides molecular understanding of directionally asymmetric catch bonding, elucidates the significance of Vcn directional catch bonding in sub-cellular organization and cellular processes, as well as describes a suite of tools for studying catch bonding mechanism in a variety of biological systems.

## INTRODUCTION

The ability of cells to generate, resist, and respond to mechanical forces applied in distinct directions is critical to many fundamental biological processes, including cytoskeletal order, directed cell migration and the developmental of anisotropic mechanical properties of tissue^1–4^. Traditionally, mechanical directional-sensitivity has often been attributed to F-actin, as it has an innately polar structure with distinct pointed and barbed ends and is often highly aligned in load-generating sub-cellular structures such as stress fibers^5,6^. However, to enable alteration or sensing of the mechanical nature of cellular microenvironment, forces must be transmitted through cellular adhesion structures. Key examples include (FAs) and adherens junctions (AJs), which respectively mediate cell-ECM and cell-cell interactions^7,8^. The overall organization of these sub-cellular structures is similar^9^. Both are composed of transmembrane receptors, integrins within FAs and cadherins within AJs, that are indirectly coupled to the force-generating actomyosin cytoskeleton through large protein plaques composed of many proteins^7^ (Fig. S1A, B) Specific multi-protein linkages that connect receptors and the actomyosin cytoskeleton have been identified in several cases ^8,10^. Prominent examples include fibronectin (FN):integrin:talin:vinculin (Vcn):actin in FAs and E-cadherin:β-catenin:α-catenin:Vcn in AJs. Thus, the ability of these multi-component linkages to maintain connectivity in response to mechanical forces is likely a primary, but poorly understood, determinant of both the bi-directional force transmission between the cell and its environment as well as the initiation of the mechanosensitive processes that regulates the assembly/disassembly of FAs and AJs^11^.

The dynamic and load bearing properties of multi-component mechanical linkages are primarily investigated by probing individual interfaces between protein pairs. The effect of applied force on bond strength between these interfaces, often measured as bond lifetime, is used to classify responses. Outside the context of adhesion biology, most interfaces behave as slip bonds, which exhibit reduced binding strength/time with increasing applied force^12^. In contrast, many of the protein interfaces in mechanical linkages within loading-bearing subcellular structures are catch bonds, exhibiting increased bond strength/lifetime with increasing applied force^13^. Interfaces between FN:α_5_β_1_ integrin, FN:α_V_β_3_ integrin, talin:actin, Vcn:F-actin, the x-dimer configuration of E-cadherin:E-cadherin, alpha-catenin:F-actin, and F-actin:myosin act as catch bonds^11,14–17^ (Supp Fig 1C). Furthermore, a substantial fraction of the interfaces with F-actin, including Vcn:F-actin, talin:F-actin, and alpha-catenin:F-actin, exhibit a directional dependence, with force applied toward the pointed end of the polar F-actin exhibiting greater catch bonding^11,16^. However, the mechanisms enabling directional catch bonding as well as the role of specific directionally-sensitive catch bonds in mediating sub-cellular structure organization and mechanosensitive cellular processes are poorly understood.

In this work, we explored the molecular basis and functions of directional catch bonding in the context of Vcn. Vcn is a highly expressed ubiquitous 117 kDa protein with three structural domains: a head domain (Vh), proline-rich linker, and a tail domain (Vt)^18^. Vcn exhibits force-sensitive localization to FAs and AJs, to regulate cell morphology, motility and force transmission^19^. Knockout of Vcn in mice causes cardiac and neural tube developmental defects and embryonic lethality at ~ E10. Mutations in Vcn are associated with muscle defects and cardiomyopathies^20^. Vcn null murine embryonic fibroblasts (Vcn^−/−^MEFs) exhibit spreading and adhesion defects, increased motility, and resistance to apoptosis and anoikis^21^. The tension across Vcn was shown to regulate a mechanosensitive switch governing the assembly/disassembly dynamics FAs^22^. Amongst catch bonds characterized at a single interface, Vcn supports the largest loads for the longest durations and exhibits substantial directional asymmetry (Fig. S1C). Previous work has shown that Vt primarily mediates both actin binding and catch bonding^11^, providing a simplified context to identify the determinants of directionally asymmetric catch bonding.

To probe the molecular mechanisms of Vcn catch bonding, we performed Discrete Molecular Dynamics (DMD) simulations of the F-actin:Vt complex subject to pulling in a variety of directions relative to F-actin. Constant pulling of Vt away from F-actin was executed in the pointed (F_P_), barbed (F_B_), and normal (F_N_) directions of F-actin. Umbrella sampling revealed that this simplified system captured key aspects of directional asymmetric catch bonds previously observed using single molecule approaches^11^. Analysis of H-bond occupancy during directional pulling simulations reveal specific residues involved in interactions that are selectively formed in response to force applied toward the pointed end of F-actin. We term these directionally asymmetric force sensitive (DAFS) residues. To assess the effects of reduced catch bonding on Vcn function, we systematically designed and constructed a set of Vt and Vcn DAFS variants. Biophysical and biochemical characterization revealed that the Vt DAFS variants retain structure, actin binding, actin crosslinking and lipid binding. To assess the effects of DAFS on Vcn function in cells, fluorescent protein-tagged Vcn as well as FRET-based biosensors for Vcn conformation^23^ and tension^24^ harboring DAFS variants were created. DAFS variants did not exhibit perturbed FA assembly or Vcn conformation, but inclusion of increasing numbers DAFS variants led to partial unloading of Vcn, as expected for reduced catch bonding. Cells expressing the DAFS variant predicted to have the least catch bonding, exhibit defects in the coordination of FAs and the actomyosin cytoskeleton, as well as an inability to undergo haptotaxis in Boyden chamber assays. Overall, this work informs the mechanistic understanding of directionally asymmetric catch bonding, elucidates the role for Vcn catch bonding in sub-cellular organization and cellular processes, as well as describes a suite of tools for studying Vcn bonding in a variety of contexts.

## RESULTS

### Molecular dynamics simulations of Vt:F-actin complex replicate direction sensitivity of Vcn catch bonding

**Figure 1 |:**
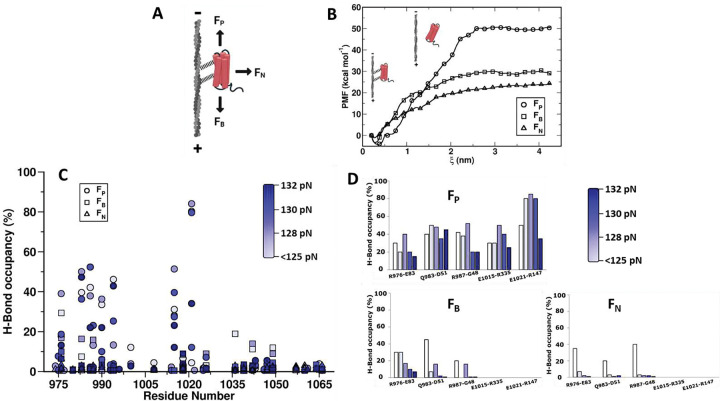
Evaluation of catch bond dynamics between F-actin and Vt. **(A)** Schematic representation of pulling forces imposed on Vt in different directions during MD simulations. **(B)** Quantification of Vt:F-actin binding strength through Potential of mean force (PMF) calculations. **(C)** Occupancy percentages of H-bond interactions between F-actin and Vt quantified from constant-force pulling discrete molecular dynamics (DMD) simulations in F_P_, F_B_, and F_N_ directions. As Vt:F-actin complexes dissociate rapidly at 150 pN with directional asymmetry and dissociation times observed in the range of 123–132 pN, H-bond occupancy percentages were depicted within this chosen range. **(D)** Comparison of DAFS interaction occupancy percentages in F_P_, F_B_, and F_N_ trajectories. Occupancy percentages for DAFS interactions at different pulling forces are shown as histograms.

Vcn engages F-actin and promotes actin filament bundling. While the actin binding determinants are contained within the Vt domain (881–1066), they are masked in the context of full-length autoinhibited Vcn^18^. Hence, the isolated Vt domain is widely used as a model for *in silico* and *in vitr*o studies^25,26^. Previous work has shown that Vcn:F-actin and Vt:F-actin interactions exhibit directionally sensitive catch bonding, with maximal stabilization observed when forces are applied toward the pointed end of F-actin (F_P_)^11^. To investigate the molecular basis for Vt:F-actin directional catch bonding properties, we performed umbrella sampling simulations^27^ on Vt:F-actin complex in F_P_, FB, and F_N_ directions (Fig. 1A). We first defined the free energy surface along a chosen coordinate to obtain directional interaction energies between F-actin and Vt, by generating one dimensional potential of mean force (PMF) curves from 26 sampling windows. When force was applied in the F_P_ direction (ΔG_P_), a larger interaction energy between F-actin and Vt (54.73 kcal/mol) was observed relative to F-actin:Vt trajectories with force acting in F_B_ and F_N_ directions (ΔG_B_ and ΔG_N_ as 33.09 and 25.02 kcal/mol, respectively) (Fig. 1B). Thus, the umbrella sampling simulations are consistent with the experimental observations of directional catch bonding of Vt with F-actin^11^ and provide support for the use of Vt as a model system to determine the primary determinants of Vcn:F-actin directional catch bonding.

### Vt forms directionally asymmetric, force strengthening (DAFS) H-bonds with F-actin.

Directional catch bonding is likely mediated by DAFS interactions that are weak or non-existent at low forces and strengthen with increasing pulling force only in certain directions. To elucidate the properties and atomistic details of DAFS interactions between Vt and F-actin, we performed constant-force pulling discrete molecular dynamics (DMD) simulations^28^ on the Vt:F-actin complex (PDB ID:3JBI)^29^. Specifically, we performed constant-force pulling of Vt by harmonically restraining the position of F-actin and applying a discretized step-function with constant energy jumps at equal distance intervals to the complex in one of three directions. To broadly characterize the system, a large set of pulling simulations were performed with forces ranging from 0–150 pN in F_P_, F_B_ and F_N_ directions and with each condition repeated ten times for a total of 4530 pulling simulations. The simulation space was considered sufficiently probed as the Vt:F-actin complex was observed to first dissociate in the range of 125 – 132 pN with dissociation occurring rapidly at 150 pN with directional asymmetry (Movies S1–S6). While the force amplitudes applied in these simulations are distinct from values observed in live cells (~2.5 pN)^24^, this is common in DMD simulations as the forces must be increased to allow feasible computational time^30^. Notably, these conditions have been shown to reflect mechanosensitive processes within physiology force regimes^30,31^.

**Figure 2 |:**
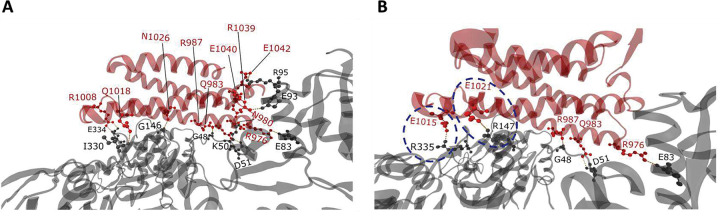
Characterization of catch bond interactions between F-actin and Vt. Visual representation of **(A)** H-bond interactions between Vt and F-actin in cryo-EM reconstruction (PDB ID: 3JBI) and **(B)** DAFS interactions for the force-loaded state of Vt:F-actin complex in the F_P_ direction. Newly induced DAFS interactions from F_P_ trajectories are enclosed in dotted circles. Color scheme: Vt is shown in red and F-actin is shown in gray. The catch bond forming residue pairs are shown in ball and stick representation.

In the cryo-EM reconstruction of Vt:F-actin complex (PDB ID: 3JBI)^29^, Vt residues in helix-4 and helix-5 bind to two adjacent actin subunits and constitute the primary unloaded binding interface. This interface has been validated using mutagenesis and F-actin binding analyses^29^, and a few mutants were shown to greatly reduce F-actin binding, including Vt I997A mutation in helix-4^32^. Residue-level contact analysis on Vt:F-actin cryo-EM reconstruction identified helix-4 residues R976, N980, Q983, R987, R1008 and helix-5 residues Q1018, N1026, R1039, E1040, and E1042 involved in H-bond interactions with F-actin residues^29^ (Fig. 2A). These key contacts were retained in DMD simulations of Vt:F-actin at 0 pN force (unloaded state), which stipulate further probing of force sensitivity and directional dependence of Vt:F-actin interactions.

To characterize key DAFS interactions between F-actin and Vt, we initially analyzed H-bond formation in the force-loaded state. H-bonds were identified by distance (acceptor–donor distance < 3.5 Å) and angle (acceptor-donor-hydrogen angle ≤ 30°) constraints, as described previously^17^. To assess the effects of applied loads, H-bond occupancy was determined as the percentage of DMD steps in a given simulation when a particular H-bond was present. H-bonds with occupancies >=40% were considered long-lived, occupancies between 20% to 40% were considered medium duration, and occupancies < 20% were considered short-lived interactions^17^. Force dependence was assessed through changes in occupancy across various simulations applied at different forces in a given direction, as previously described^17^. Directional asymmetry was detected by comparing force-sensitive changes in occupancy percentages across different pulling directions.

When forces were applied in the F_P_ direction, 34 H-bonds were detected between Vt and F-actin. Five were long-lived, 3 were of medium duration, and 26 were short-lived. Out of 26 short-lived H-bonds, 7 were weakened with increased pulling force and 19 were lost (ruptured). All medium duration interactions (E986/G48, T990/G46, and Q994/Y143) were newly formed and subsequently weakened with increased pulling force. Interestingly, all five long-lived interactions were strengthened (increased occupancy) by force (125–128 pN) (Fig. 1C and 1D). Three of these long-lived interactions (R976/E83, Q983/D51, R987/G48 Vt/F-actin) exist in the unloaded state but become stronger with increased force. The other two formed new H-bond interactions (E1015/R335, E0121/R147 Vt/F-actin) induced between helix-5 of Vt and F-actin protomer and subsequently reinforced with an increase in pulling force (Fig 1D). Beyond the pulling force of 128 pN, the DAFS interactions in the F_P_ direction were weakened and eventually resulted in the separation of the Vt:F-actin complex. This is consistent with the expected catch-slip transition of realistic bonds^11,17^.

During force application in the F_B_ direction, 20 H-bonds were identified between Vt and F-actin. While no long-lived interactions were evident, two medium duration and 18 short-lived interactions were observed between Vt and F-actin. Out of two medium duration interactions, one was newly formed (E1036/K50 Vt/F-actin) and the other (R976/E83 Vt/F-actin) was present in the unloaded state. Both of these medium duration interactions lost their strength with increased pulling force. Interestingly, five out of 18 short-lived H-bonds (Q983/D5, R987/G48, Q1018/A331, N1026/G146, and E1042/R95 Vt/F-actin), exist in the unloaded state, were marginally strengthened in the force range of 125 – 128 pN (Fig. 1C). The remaining 13 short-lived interactions were weakened with increased pulling force. Beyond the pulling force of 128 pN, all interactions displayed slip bond characteristics until rupture. Interactions between that amino acids pairs that formed long-lived interactions induced by pulling in the F_P_ direction (E1015/R335, E0121/R147 Vt/F-actin) (Fig. 2B) were not evident in simulations of pulling in the F_B_ direction.

Loading in the F_N_ direction revealed 21 H-bond interactions. All these interactions were short-lived with occupancy percentages below 10% (Fig. 1C). Eight of the 21 were induced H-bonds, and the remaining 13 were present in the unloaded state. None of the 21 short-lived interactions were strengthened with increased pulling force. The occupancy percentages of all Vt:F-actin interactions gradually reduced with increase in pulling force and eventually resulted in the rapid detachment of Vt from the F-actin surface. Also, interactions between that amino acids pairs that formed long-lived interactions induced by pulling in the F_P_ direction (E1015/R335, E0121/R147 Vt/F-actin) (Fig. 2B) were not evident in simulations of pulling in the F_N_ direction (Fig. 1D).

Altogether, these analyses demonstrate that simulations of the Vt:F-actin complex are suitable for studying catch bonding of Vcn:F-actin as well as reveal the atomistic interactions mediating directional asymmetry and force-dependence. Notably, drastically different H-bonds were formed in the various pulling directions, with maximal H-bond stabilization occurring in F_P_ direction due to an alteration in the Vt:F-actin interface (Fig. 2B). The stabilization under load in the F_P_ direction is due to a combination of strengthened as well as induced H-bonds. Long-lived, strengthened H-bond contacts are found mainly in helix-4 residues. These include the guanidino sidechain of R976 (Vt) and the side chain carboxyl group of E83 (F-actin), the sidechain amino group of Q983 (Vt) and the side chain carboxyl group of D51 (F-actin), and the sidechain amino group of R987 (Vt) and the backbone carboxyl group of G48 (F-actin) (Fig. 2B). Long-lived, induced H-bonds are formed by E1015 (Vt) and E1021 (Vt) as helix-5 repositions due to force application in the F_P_ direction (Fig. 2B). Specifically, the side chain carboxyl of E1015 (Vt) interacts with the side chain amino group of R335 (F-actin) and the side chain carboxyl group of E1021 (Vt) forms an H-bond interaction with the side chain amino group of R147 (F-actin) (Fig. 2B). Importantly, H-bond interactions involving E1015 and E1021 have particular high occupancies under loaded and only form with loading in the F_P_ direction. Therefore, it is likely these DAFS H-bonds are particularly relevant in mediating directional catch bond formation by Vcn.

### Creation of a suite of DAFS Vt variants

To optimally identify mutations that perturb F_P_-specific DAFS interactions between Vt and F-actin yet retain Vt structure, we performed *in silico* mutagenesis of E1015 and E1021 using the Eris molecular modeling suite^33^. Eris employs fast side-chain packing and backbone relaxation algorithms to quantify the change in protein structural stability upon mutagenesis^33^. *In silico* computational mutagenesis using the Eris molecular modeling suite predicted that substitution of alanine for E1015 and E1021 represent stabilizing mutations (ΔΔG < 0) that retain Vt structure and Vt: F-actin interactions in the unloaded state. Eris calculations on the E1015A-E1021A double mutant were also predicted to retain Vt structure and F-actin interactions in the unloaded state. Hence, we chose three DAFS variants (E1015A, E1021A, and E1015A-E1021A) for *in vitro* structural and biochemical studies.

### DAFS variants do not perturb Vt structure and stability

**Figure 3 |:**
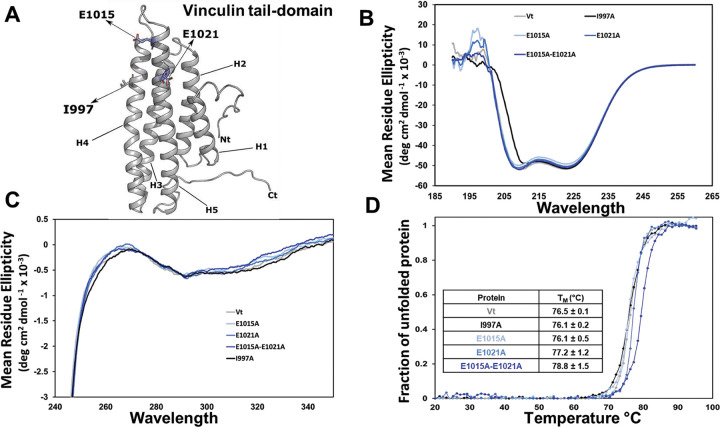
Mutation of DAFS residues retains Vt structure and stability. **(A)** Ribbon diagram of Vt with potential DAFS residues highlighted in blue. Isoleucine 997 (grey licorice) in Vt is a key actin binding residue. **(B)** Far-UV CD spectral profiles of DAFS variants. **(C)** Near-UV spectral overlay of DAFS variants. **(D)** Melting temperature (T_M_) of DAFS variants calculated from CD thermal melt curves. Representative CD data are shown from three independent experiments. The T_M_ melt curves are presented as an average of 3 scans from 3 independent experiments. Error in T_M_ is shown with standard errors.

To evaluate the contribution of DAFS residues in maintaining the conformation and stability of Vt (Fig. 3A), we performed far-ultraviolet (UV), near-UV and circular dichroism (CD) thermal melt profiling of DAFS variants containing alanine substitutions. All Vt DAFS variants showed similar far-UV CD spectral profiles, indicating minimal effects on secondary structure relative to WT Vt (Fig. 3B). We also compared the near-UV spectra of the DAFS variants to evaluate tertiary packing interactions between the N- and C-terminus, as Vt has a distinct near UV pattern between 260–300 nm (positive/dip) due to tertiary packing of two tryptophan residues located at W912 in the H1/H2 loop and W1058 in the C-terminus^34^ (Fig. 3C). The near-UV spectral profile associated with the single (E1015A), (E1021A), and double (E1015A-E1021A) Vt DAFS variants were similar to WT Vt, suggesting that the DAFS variants possess similar tertiary structure (Fig. 3C). Moreover, similarity in the CD melt curves relative to WT Vt indicates that the mutations do not alter thermal stability (Fig. 3D). These data reveal that the single and double DAFS variants do not exhibit altered conformation or stability of Vt.

### DAFS variants retain Vt-F-actin binding and bundling

**Figure 4 |:**
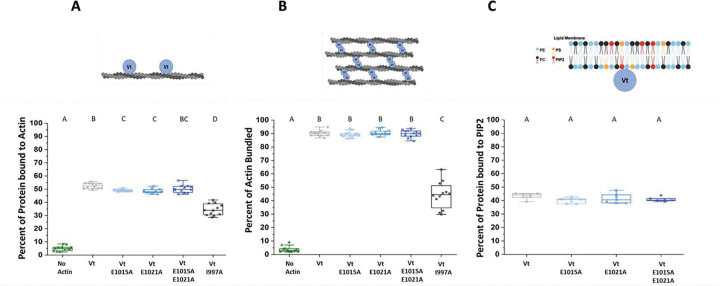
DAFS variants retain Vt interactions with F-actin and PIP_2_. **(A)** High speed F-actin co-sedimentation assays comparing actin binding of WT Vt, DAFS and the actin-deficient I997A Vt variant. Vt I997A is an actin binding deficient control. Box-whisker plot with 12 data points from 3 independent experiments are presented. **(B)** Low speed F-actin co-sedimentation assays with WT Vt, DAFS and Vt I997A variants comparing the fraction of F-actin present in bundles or in higher-order assemblies. **(C)** Box-whisker plot with 7 data points from 2 independent experiments comparing WT Vt and DAFS Vt variant association with PIP_2_ containing LUVs. One-way analysis of variance (ANOVA) and Tukey’s HSD test were used for statistical analysis. Different letters denote significant difference at p<0.05. See Table S1 for a detailed listing of p-values.

To probe the effects of DAFS residue mutation on Vt:F-actin binding, we conducted high speed actin binding co-sedimentation assays using WT Vt as a positive control and Vt I997A as actin binding deficient variant (Fig. 4). We find that the double (E1015A-E1021A) DAFS Vt variant binds F-actin similarly to WT Vt (Fig. 4A), and a small reduction (2%) was observed for the E1051A and E1021A variants. As binding of F-actin promotes Vt dimerization and F-actin crosslinking, we also quantified F-actin bundling of the Vt DAFS variants using low-speed actin co-sedimentation assays. We find that single (E1015A, E1021A) and double (E1015A-E1021A) Vt variants can bundle actin similar to WT Vt (Fig. 4B). While E1015A and E1021A DAFS variants exhibit slightly lower (~2%) F-actin binding relative to WT Vt, the actin-bundling ability of both single variants remained unperturbed suggesting the slight reduction in actin binding is functionally insignificant. Overall, our biochemical data show that the single and double DAFS Vt variants do not perturb interactions with actin.

### DAFS variants retain Vt-PIP_2_/lipid interactions

Vcn specifically associates with the acidic phospholipid, phosphatidylinositol-4,5-bisphosphate (PIP_2_), to drive association with the membrane. To determine whether the DAFS variants affect association with 100 nm large unilamellar vesicles (LUVs) containing phosphocholine (PC), phosphoethanolamine (PE), and 20% phospho-L-serine (PS) and 10% PIP_2_, we conducted lipid co-sedimentation studies^25,34^ (Fig. 4C). Our results indicate that Vt single and double DAFS variants retain PIP_2_ specificity and exhibit strong association with anionic PIP_2_ containing LUVs similar to WT Vt (Fig. 4C).

### DAFS variants do not perturb FA morphometrics

**Figure 5 |:**
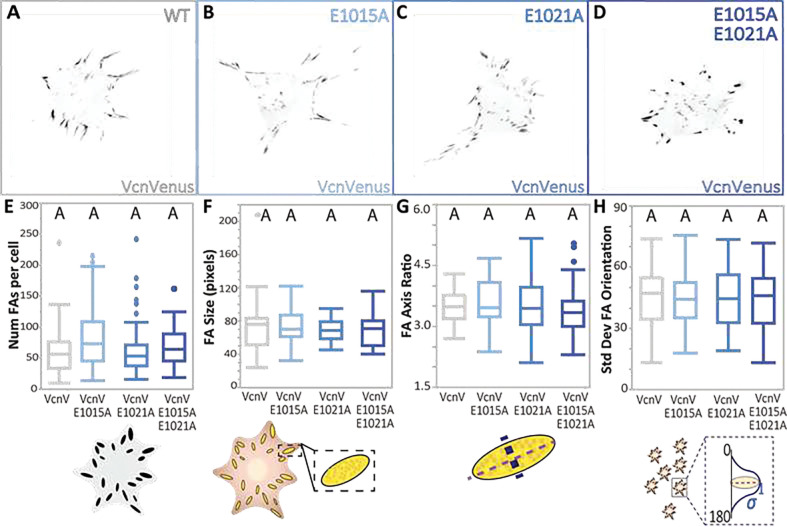
Vcn DAFS variants do not display altered FA characteristics. **(A-D)** Representative images of Vcn^−/−^ MEFs expressing WT VcnVenus or DAFS variants of VcnVenus. WT VcnVenus is shown in gray and DAFS variants of VcnVenus are shown in shades of blue. Quantification of FA morphometric characteristics including (**E)** FA number, (**F)** FA area, (**G**) FA axis ratio, and (**H**) standard deviation of FA orientation. Plots shown for WT VcnVenus and DAFS variants of VcnVenus (*n* = 45, 52, 59, 51 cells, respectively, collected over 3 independent experiments). One-way ANOVA paired with a Steel-Dwass test (E, F, G) or a Tukey’s HSD test (H) conducted across all VcnVenus, VcnCS and, VcnTS constructs were used for statistical analyses. Differing letters denote significant difference at *p*<0.05. See Table S1 for a detailed listing of p-values.

Next, we sought to evaluate the effects of the DAFS variants on Vcn in cells. Constructs encoding single and double DAFS Vcn variants tagged with the fluorescent protein mVenus (VcnVenus) were created and transiently expressed in Vcn^−/−^ mouse embryonic fibroblasts (MEFs). To determine effects on FA initiation, FA maturation, FA elongation, and overall FA organization, the number of FAs per cell, FA size, FA major axis to minor axis ratio, and standard deviation in FA orientation were quantified. As shown in Fig. 5, FA morphometrics quantified at matched expression levels (determined by local Venus intensity) did not change with the introduction of DAFS Vcn variants. This is consistent with previous work demonstrating FA morphometrics remain similar across cells expressing WT Vcn or the actin binding deficient Vcn I997A^32^.

### DAFS variants do not affect Vcn Activation

Vcn is subject to classical head-tail inhibition^35^. Dysregulation of the interface that mediates this interaction can lead to activation of Vcn and excessive accumulation of Vcn within FAs^36^. A FRET-based biosensor incorporating one fluorescent protein in the strap region of Vcn and another at the C-terminus reports Vcn conformation, both within FAs and the cytoplasm^23,24^. To determine if DAFS Vcn variants affect activation of Vcn, Vcn conformation sensors (VcnCSs) harboring none, one or both variants were created and transiently expressed in Vcn^−/−^ MEFs. All VcnCS variants localized to FAs as expected, and FA morphometric characteristics were consistent with respective VcnVenus variants (Fig. S2). Furthermore, FRET efficiencies within the cytosol were similar for all variants and consistent with estimates of closed Vcn (Fig. S4 and S5). At FAs, FRET efficiencies for all DAFS VcnCS variants were not different, and consistent with the presence of active Vcn (Fig. S7). Together, these data demonstrate that the DAFS variants do not affect Vcn conformation.

### DAFS variants progressively, but incompletely, unload Vcn

**Figure 6 |:**
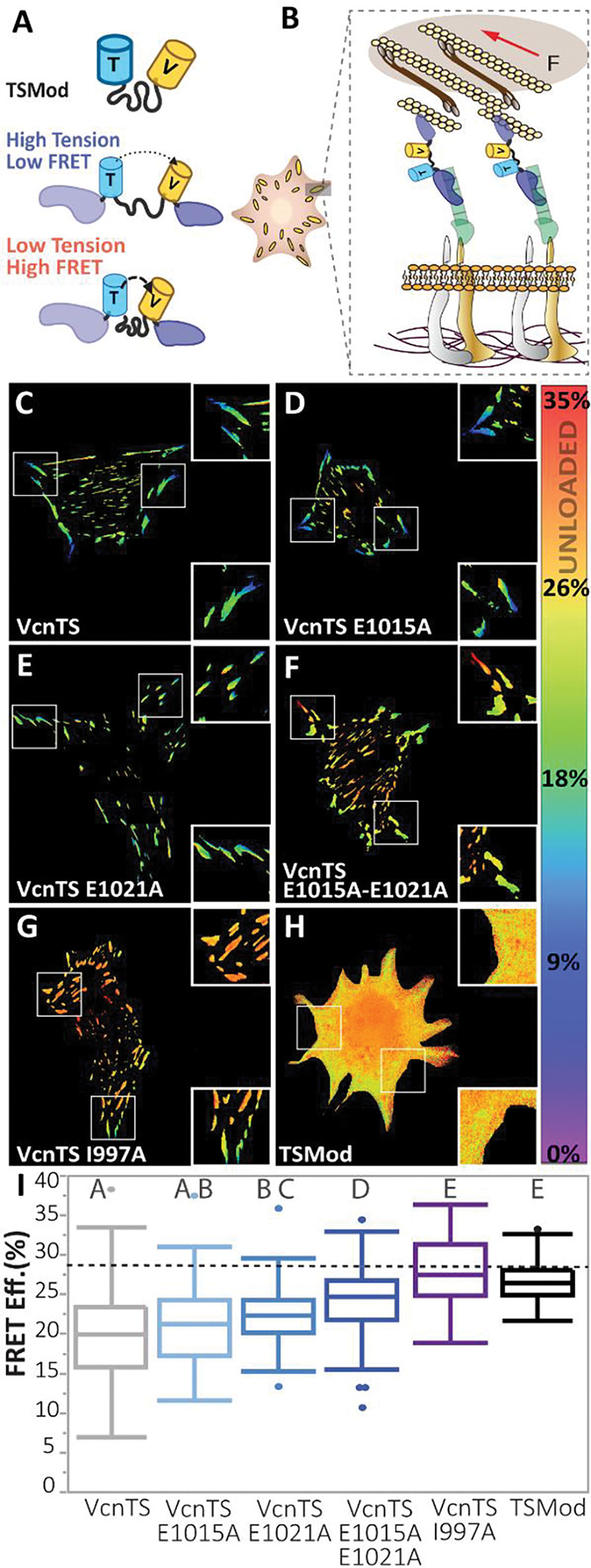
DAFS variant VcnTSs experience reduced, but not negligible, load **(A)** Schematic of TSMod and VcnTS constructs with loaded and unloaded states. **(B)** Schematic of VcnTS in the FA, loaded by actomyosin contractility. **(C-G)** FA masked FRET efficiency is shown for single Vcn^−/−^ MEFs expressing WT VcnTS, DAFS variants of VcnTS and a previously validated variant with reduced actin affinity, VcnTS I997A (*n* = 232, 85, 79, 97, 58 respectively, pooled from 11 days). (**H)** Representative cytosolic FRET efficiency is shown for a single Vcn^−/−^ MEF expressing unloaded FRET control, TSMod (*n*= 69, from individual experimental 5 days). **(I)** Box-whisker plots are shown for cell-averaged FRET efficiency of VcnTS and DAFS variants of VcnTS compared to expected unloaded FRET efficiency (dotted line) controls. One-way ANOVA and a Steel-Dwass test were used for statistical analysis. Differing letters denote significant difference at *p*<0.05. See Table S1 for a detailed listing of p-values.

The engagement of a catch bond is thought to enable the support of larger mechanical loads^11^. Therefore, we sought to determine the effects of DAFS variants on the loads supported by Vcn. A FRET-based biosensor incorporating two fluorescent proteins and an extensible domain within the Vcn strap region has previously been used to report mechanical loading of Vcn in diverse contexts^24,37,38^ (Fig. 6A and 6B). To determine if DAFS variants affect the loads experienced by Vcn, Vcn tension sensors (VcnTSs) harboring none, one or both DAFS variants were created and transiently expressed in Vcn^−/−^ MEFs. An actin-binding deficient VcnTS I997A was used as an unloaded control^32^. All DAFS VcnTS variants localized to FAs as expected, and the FA morphometric properties were consistent with the respective VcnVenus variants (Fig. 8). Remarkably, with the successive inclusion of DAFS variants, Vcn load was reduced at FAs (Fig. 6C–F and 6I). Within the cytoplasm, FRET efficiencies for all variants were similar and consistent with estimates of unloaded Vcn (Fig. 9). Furthermore, VcnTS I997A appeared unloaded, as the observed FRET efficiency was on par with tension sensing module (TSMod), the cytosol-expressed tension sensing module without flanking Vcn domains^39,40^ (Fig. 6G, H, and Fig. S9). The support of an intermediate level of loading is expected of mutations that eliminate catch bonding, but not Vcn:F-actin interactions. Thus, this data shows that DAFS residues contribute to the ability of Vcn to bear load and is consistent with DAFS residues mediating Vcn catch bonding.

### DAFS residues mediate co-alignment of SFs and FAs

**Figure 7 |:**
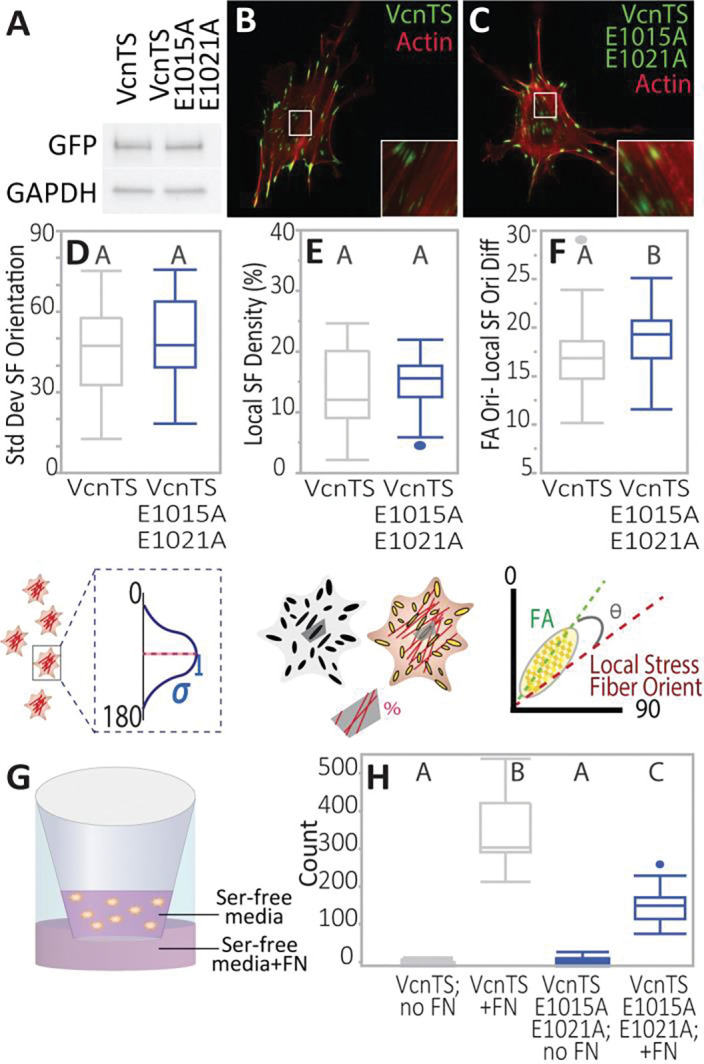
Local FA-SF alignment and directed migration ability differ between WT VcnTS and DAFS double variant VcnTS. **(A)** Western of stably expressed VcnTS and DAFS double variant VcnTS E1015A-E1021A cell lines, showing presence of mutations does not affect VcnTS cellular expression ability. Anti-GFP used to label VcnTS. **(B)** VcnTS or **(C)** DAFS double variant VcnTS E1015A-E1021A stained for F-actin. Box-whisker plots quantifying **(D)** spread of actin SF orientations **(E)** SF density per FA-defined local region, and **(F)** angle between FA and average angle of actin filaments within respective Voronoi regions of cells. Plots shown for stably expressed VcnTS or VcnTS E1015A-E1021A in Vcn^−/−^ MEFs (*n* = 36, 45 respectively, from three independent experimental days). Differing letters denote significant difference at *p*<0.05, Student’s t-test followed by post-hoc Tukey test (C, D) and Steel-Dwass test for unequal variances (E). **(G)** Schematic depicting transwell setup for directed migration assay. **(H)** Box-whisker plots are shown of migrated cells per field of view (FOV) in a Boyden chamber haptotaxis migration assay (*n*= 19,18,16,18 FOVs per given group from three independent experiments). One-way ANOVA and a Steel-Dwass were used for statistical analyses. Differing letters denote significant difference at *p*<0.05. See Table S1 for a detailed listing of p-values.

Simulations have suggested that the directional catch bond between Vcn and F-actin mediates the coordination of FAs and the actin cytoskeleton^11^, but experimental data are lacking. Therefore, we sought to probe the role of DAFS residues in the coordination of these subcellular structures. Efforts focused on double variant constructs as these had the largest loss of loading, short of complete unloading (Fig. 7). To begin, Vcn^−/−^ MEFs stably expressing either WT VcnTS or VcnTS E1015A-E1021A (Fig. 7A) were stained for F-actin with phalloidin (Fig. 7B and 7C). The standard deviation of cellular stress fiber (SF) orientations was quantified to assess actin organization (Fig. 7D). No differences were found between VcnTS and VcnTS E1015A-E1021A expressing cells. Next, we sought to probe the coordination of FAs and the actomyosin cytoskeleton. To do so, a Voronoi tessellation was used to define local FA-centered regions within the cells. Within each region, local SF density was calculated and no differences were observed (Fig. 7E)^41^. Next, vectors corresponding to the local orientation of the FA (obtained by blob analysis) and the local orientation of SFs were generated (Fig. S8). The difference of these vectors was taken as a measure of the coordination between the FA and the actomyosin cytoskeleton, with a smaller difference indicative of increased coordination. VcnTS E1015A-E1021A expressing cells showed reduced co-alignment compared to VcnTS expressing cells (Fig. 7F), consistent with a key role for DAFS residues in mediating coordination of FAs and the actomyosin cytoskeleton.

### DAFS residues are required for optimal haptotaxis

Vcn is a critical mediator of directional migration^42,43^. To determine if DAFS residues are important in directed cell migration, Vcn^−/−^ MEFS expressing either WT VcnTS or VcnTS E1015A-E1021A at equivalent levels (Fig. 7A) were subject to a Boyden chamber based haptotaxis assay (Fig. 7G). Nearly double the number of VcnTS MEFs underwent haptotaxis, compared to the VcnTS E1015A-E1021A MEFs (Fig. 7H). Thus, DAFS residues, and likely the engagement of the Vcn:F-actin catch bond, promotes directed cell migration^11^.

## DISCUSSION

The ability of cells and tissues to resist or deform differentially in response to mechanical forces applied in distinct directions is mediated by the ability of load-bearing proteins to maintain or change connectivity in response to these forces^44,45^. Previous work has identified several key examples, and simulations have predicted key roles for directional catch bonds in the polar or directed molecular and cellular process^11,16^. To test these predictions, we developed a procedure for *in silico* identification, rational engineering, biochemical validation, and probing the *in cellulo* roles of amino acids mediating directional catch bonding. Specifically, we employed a combination of molecular dynamics simulations, *in silico* mutagenesis, controlled biochemical assays, FRET-based biosensors, quantitative image analysis, and cell migration assays to fully characterize the ability of the mutation of identified amino acids to perturb catch bonding, minimally affect other functions, and determine the resulting changes in cell sub-structures and cellular behavior.

We focused on the mechanical linker protein Vcn, as it mediates force transmission in multiple subcellular structures and exhibits the strongest directionally sensitive catch bond described to date^11^. Specifically, single molecule force measurements showed that Vcn forms directionally sensitive catch bonds with F-actin, with maximal stabilization (~10-fold increased mean bond time) when forces are applied in the F_P_ direction compared to the F_B_ direction^11^. However, experimental characterization of catch bonds in the F_N_ direction was not performed. Here, we employed umbrella sampling simulations to quantify directional force-dependent interactions between Vt and F-actin. Consistent with experimental observations^11^, we found that Vt engages F-actin most strongly with force applied in the F_P_ direction, consistent with previous experimental observations^11^. These predictive computational findings provided a solid basis to further explore the molecular mechanism of DAFS interactions between Vt and F-actin using constant-force pulling DMD simulations in F_P_, F_B_, and F_N_ directions. While forces in the F_P_ direction induced a variety of long-lived H-bonds that either strengthened or formed under load, forces in the F_B_ direction induced medium duration bonds. These differences likely underlie experimentally-observed directional catch bonding between Vcn and F-actin (Fig. S1)^11^. In the F_N_ direction, only short-lived interactions were observed, suggesting a limited ability to resist applied loads.

Based on analyses of the DMD simulations and the Vt/F-actin complex structure, we posit a simple working model (Fig. S12) of directional catch bonding in Vcn. Overall, catch bonding seems to be due to a combination of force-induced structural changes in Vt and the asymmetric nature of F-actin. Specifically, F_P-_directed pulling force induces a twisting rearrangement of Vt helical bundle along the short-axis of the actin filament that exposes side chains of E1015 and E1021 (Fig. S11). This generates a new binding interface at the C-terminal end of helix-5 to form H-bond interactions with F-actin residues R335 and R147, as well as reinforce some existing H-bonds in helix-4 (R976/E83, Q983/D51, R987/G48 Vt/F-actin) through improved alignment of side chain orientations. The twisting of Vt as well as the strong interactions are not observed when Vt is pulled in the F_B_ direction. Therefore, distinctly weaker H-bonds are formed, and existing H-bonds are strengthened to a lesser degree (Fig. S12), likely due to the structural heterogeneity and intrinsic polarity (the direction of relative translocation) of F-actin^46^. Only weak, short-lived interactions were observed in the F_N_ trajectories, possibly due to the orthogonal pulling of Vt away from the actin filament (perpendicular to the F-actin surface) (Fig. S12).

Through identification and manipulation of key DAFS interactions that were uniquely induced by forces in the F_P_ direction, we created a variety of mutations to prevent directional catch bonding. We sought to identify DAFS variants that retain Vt structure as well as F-actin and phospholipid interactions. We first performed structural analysis using near, far, and temperature-dependent CD measurements and found that the DAFS variants do not alter Vt structure. We then examined the ability of Vt DAFS variants to bind and bundle F-actin *in vitro* using a set of actin co-sedimentation assays. Also, lipid co-sedimentation assays were conducted to assess maintenance of binding strength and binding specificity to phospholipids in the Vt DAFS variants. DAFs variants exhibited similar F-actin binding, bundling, and lipid recognition relative to WT Vt, indicating retention of these key binding interactions and justifying the use of DAFS variants to study Vcn directional catch bonding.

To assess the roles of directional catch bonding on vinculin function *in cellulo*, DAFS mutations were incorporated into Vcn-Venus and Vcn biosensors for studying the interplay of Vcn directional catch bonding, Vcn activation, and Vcn loading. FA shape was unaffected by the loss of catch bonding activity, broadly consistent with previous work involving the weakening of Vcn:F-actin interactions^47^. As expected, Vcn loading was reduced by loss of Vcn catch bonding but regulation of Vcn conformation was unaffected^47^. Vcn assumes a closed, autoinhibited conformation mediated by a strong interaction between the head and tail domain that must be relieved to enable opening and interaction with key binding partners, such as F-actin^35^. A variety of models have been developed to explain this process with key aspects being the co-incidence of multiple activated binding domains, regulation by post-translational modification, as well as direct opening of Vcn through the application of tension^18,48^. The data collected here are most consistent with a model where Vcn conformation and Vcn load are independently regulated and inconsistent with models suggesting that Vcn loading “pulls open” Vcn to promote activation^24,49^. A key question for the future will be determining the specific regulators of these distinct processes.

Next, we sought to probe the effects of loss of Vcn directional catch bonding on adhesion-associated, mechanosensitive sub-cellular and cellular processes. We first evaluated the relationships between FAs and SFs, as these change during adhesion maturation and catch bonds have been proposed to play a critical role^18,36,38,50,51^. Like the physical characteristics of the FAs (e.g. size, elongation, or orientation), stress fiber assembly was unperturbed upon introduction of both DAFS variants to Vcn. Furthermore, cells expressing this double DAFS variant Vcn showed defects in the co-alignment of FAs and F-actin, consistent with the prediction that directional catch bonds are important in mediating local F-actin alignment^11,16^. These data are consistent with a model where other mechanisms dictate the assembly of FAs and SFs, but Vcn directional catch bonding mediates coordination of these sub-cellular structures^52^. Interestingly, a recently developed mathematical model suggests that the self-stabilization of linker proteins (e.g. Vcn binding to unfolded talin domains and preventing re-folding), is the dominant driver of adhesion strengthening^53^. A key issue for future work is further elucidating the roles of self-stabilization and directional catch bonding in mediating the coordinated assembly and organization of FAs and stress fibers. To probe a cellular process, we focused on directed migration in response to a haptotactic gradient. We found that Vcn directional catch bonding specifically is required for efficient directed migration, broadly consistent with our previous work demonstrating the importance for Vcn:F-actin interactions in directed cell migration^38^. This result establishes a key role for Vcn catch bonding in a fundamentally important cellular process. Overall, these data suggest that, while most studies have focused on the assembly dynamics of FAs and stress fibers individually, the coordination of these processes may be particularly relevant for studying directed cell migration.

To assess the generalizability of our results, we compared Vcn and α-catenin (Catn), as they are structural homologs that are both recruited to AJs in response to applied loads and exhibit directional catch bonding with F-actin^54,55^. Cryo-EM reconstructions of F-actin bound Catn (PDB ID: 6UPV)^54^ and Vt (PDB ID: 3JBI)^29^ show similar engagement to F-actin (RMSD = 0.99 Å,), with key catch bond residues in Vt residues similar in orientation to Catn with respect to the actin interface. Specifically, M816 of Catn was predicted to form force-sensitive catch bond interactions with F-actin^55^, which sequentially and structurally align with E1021 of Vcn. Another noteworthy comparison is between D813 of Catn and E1015 of Vcn, which point in the same direction to induce catch bonds with F-actin. Since D813 of Catn and E1015 of Vcn are charged residues with identical physicochemical properties, they can induce catch bond interactions of similar strengths. However, the corresponding residue to Vcn E1021 is M816 in Catn. Catch bond formation with this non-polar residue will likely be weaker relative to Vcn. Given these observations, we postulate that even though both Vcn and Catn induce similar directional and force-dependent interactions with F-actin, the strength of Catn:F-actin interactions are weaker than Vt:F-actin catch bonds (Fig. 1C and Fig. 2B).

Overall, this work develops and implements an interdisciplinary approach for identifying and perturbing directional catch bonding between F-actin and Vcn. While cell-ECM adhesion and single migration were probed here, Vcn also has key roles is cell-cell adhesion, collective cell migration, and a variety of developmental as well as pathophysiological processes^56^. Furthermore, this approach will likely be generalizable to other F-actin binding loading-bearing proteins and enable assessment and potential manipulation of directional catch bonding in this large class of proteins. Additionally, catch bonds mediate diverse processes such as bacterial adhesion^57^, binding of neutrophils to inflamed endothelial cells^58^ and T-cell receptor activation^59^. The procedure for identifying key residues and producing tools to probe the effects of the loss of directional catch bonding developed here will facilitate a plethora of studies in mechanobiology, at the molecular, cellular, and eventually tissue-level across these wide-ranging fields.

## MATERIALS AND METHODS

### Umbrella sampling simulations to quantify the interaction energy between F-actin and Vt

To generate a series of F-actin–Vt structural conformations with increased center-of-mass (CoM) distance between F-actin and Vt, we considered the cryo-EM structure F-actin:Vt (PDB ID:3JBI)^29^ and pulled Vt away from the actin filament along F_P_, F_B_, and F_N_ directions separately (Fig 1a). During the pulling simulations, position restraints were applied on backbone heavy atoms of F-actin to keep it immobile and no position restraints were applied on Vt. In each direction, the pulling simulations were executed for 1000 ps, using a pull rate of 0.01 nm/ps and a spring constant of 1000 kJ/mol/nm^2^. From each pulling trajectory, snapshots of F-actin–Vt were extracted and utilized as starting configurations for the umbrella sampling windows^27^. Each of these configurations corresponds to harmonically restrained Vt away from F-actin, via an umbrella biasing potential. The harmonic restraint between F-actin and Vt allow Vt to sufficiently explore the configurational space in each window along a reaction coordinate (ξ)^27^. We chose a window spacing between 1 – 20 Å CoM separation between F-actin and Vt, which resulted in 26 windows in each pulling trajectory. The actin-Vt configuration in each window was solvated with water molecules, and 150 mM NaCl was added to neutralize the charge of the system. We chose the CHARMM36 forcefield^60^ to generate bonded and non-bonded interaction parameters of all atoms in the system, and performed steepest descents energy minimization followed by position restraints energy minimization for 100 ps under constant-temperature, constant-volume (NVT) ensemble. During the equilibration, protein and non-protein atoms were separately coupled to temperature baths and maintained at a temperature of 310K using the Berendsen weak coupling method ^61^. Subsequently, we carried out 100 ps of equilibration in constant-temperature, constant-pressure (NPT) ensemble to maintain isotropic pressure at 1.0 bar using the Parrinello-Rahman barostat^62^. Finally, we employed Nosé- Hoover thermostat^63,64^ and the Parrinello-Rahman barostat^62^ for a 10 ns production run in each window with position restraints on F-Actin. We calculated long-range electrostatics using the particle mesh Ewald (PME) algorithm ^65^ and chose the cut-off of short-range nonbonded interactions at 1.4 nm. All MD simulations were conducted using the GROMACS-2018 package^66^. We derived potential of mean force (PMF) from each of the simulations, assembled the PMF curve with respect to the reaction coordinate, and estimated the binding energy (ΔG) between F-actin and Vt in three pulling directions using the weighted histogram analysis (WHAM) method^67^.

### Constant-force pulling discrete molecular dynamics (DMD) simulations to identify force-dependent interactions between actin and Vcn

The 8.50 Å cryo-EM reconstruction of the F-actin/Vt complex (PDB ID: 3JBI)^29^ was used as a starting structural model for constant-force pulling simulations. The missing N- and C-terminal residues of Vt were added by homology modeling using the high-resolution X-structure of Vt (PDB ID: 1QKR)^68^ as the template. The best F-actin–Vt complex model with optimized modeler objective function was selected out of forty generated models using Modeller-9v19^69^. The optimized structure was placed in a cubic box with side-length 500 Å with periodic boundary conditions. The Anderson thermostat was used to maintain temperature at 300 K (room temperature). Constant-force pulling was achieved by applying a discretized step-function with a constant energy jump, dE, at the distance step of dR (1 Å) between F-actin and Vt, where the pulling force (f) is estimated as dE/dR, with dE of approximately 0.015 kcal/mol^30^. Constant pulling of Vt away from F-actin was executed in F_P_, F_B_, and F_N_ directions by employing a discretized step-function with a constant energy jump at equal distance intervals. The position of F-actin was harmonically restrained during the pulling simulations. Based on previous pulling simulations on biological complexes^30^, we chose pulling forces within range 0–150 pN and sampled at an interval of 1 pN. Each pulling simulation at a specific force was carried out for 400,000 discrete molecular dynamics (DMD) steps and repeated 10 times to establish the confidence interval. The data obtained from our simulations was subsequently used to analyze the directionally asymmetric, force strengthening (DAFS) h-bonds between F-actin and Vt. Data from simulation trajectories was extracted using in-house scripts and Gromacs analysis tools. The representative 3D conformations of F-actin and Vt were rendered using PyMol^70^ and visual molecular dynamics (VMD)^71^.

To determine H-bond formation between donor (D) and acceptor (A), we employed a cut-off distance of 3.5 Å with the acceptor-donor-hydrogen angle ≤ 30°^17^ and subsequently quantified the occupancy of each H-bond interaction over the entire MD trajectory^72^. H-bond occupancy is defined as a fraction of conformations in which a specific residue pair is involved in H-bond interactions. We converted these occupancy fractions to occupancy percentages by multiplying with ‘100’.

### Eris calculations to identify suitable benign catch bond mutations for experimental evaluation

*In silico* mutagenesis studies on Vt DAFS residues were performed using Eris molecular suite^33^. The high-resolution X-structure of Vt (PDB ID: 1QKR)^68^ was used to evaluate residue perturbations. Initially, Eris employs residue substitutions in protein structure and evaluates free energies of native (ΔG_wt_) and mutant (ΔG_mut_) conformations. Then, Eris computes the change in free energy of protein upon mutation (ΔΔG_mut_) by subtracting the free energy of native protein from that of mutant. Based on ΔΔG_mut_ values, Eris estimates mutations as either stabilizing (ΔΔG_mut_ < 0) or destabilizing (ΔΔG_mut_ > 0).

### Plasmid Design

For stability and interaction studies, Vt (comprising the chicken sequence 879–1066) was cloned into a pQlinkH vector (Addgene, Cambridge, MA). All DAFS Vt variants, E1015A, E1021A, and E1015A-E1021A were constructed using a Q5 site-directed mutagenesis kit (New England Biolabs, Ipswich, MA). For cellular studies, variants of VcnVenus and variants of VcnTS plasmids were constructed using appropriate primers (Eton Biosciences, Durham, NC) and Q5 site-directed mutagenesis (New England Biolabs, Ipswich, MA) on the pcDNA 3.1 Vcn Venus (Addgene Plasmid #27300) and the pcDNA3.1 VcnTS plasmid (Addgene Plasmid #26019), respectively. pcDNA 3.1 VcnCS with mTFP1-Venus(A206K) fluorescent protein was cloned as described previously^23,24^. Site-directed mutagenesis on the pcDNA3.1 VcnCS plasmid was also used to generate variants of VcnCS. pRRL VcnTS (Addgene Plasmid #111830) and variants of pRRL VcnTS were generated via 5′*Nru*I/3′*Xba*I digestion and ligation of the Vh-TSMod-Vt sequence from pcDNA 3.1 VcnTS (T4 DNA Ligase; New England BioLabs, Ipswich, MA) into a pRRL vector digested with 5′*Eco*RV/3′*Xba*I. All constructs were verified via DNA sequencing (Azenta Life Sciences, Morrisville NC).

### Protein Expression and Purification

All pQlinkH expression vectors contain an N-terminal His-tag and TEV cleavage site. WT Vt domain and Vt DAFS variants were expressed in the Escherichia coli BL21 (DE3) strain and purified as previously described^73^. Briefly, cells were first grown at 37 °C to an optical density of 0.6–0.8 at 600 nm. Protein expression was then induced by addition of isopropyl-D-1-thiogalactopyranoside (0.5 mM). After induction, cells were grown overnight at 18 °C and harvested by centrifugation at 4500 rpm for 30 minutes. Cell pellets were resuspended in lysis buffer (20 mM Tris, 150 mM NaCl, 5 mM imidazole, 2 mM β-mercaptoethanol, pH 7.5), lysed by sonication and purified first by affinity separation using Ni-NTA-agarose beads (Qiagen). Vt protein bound to the His-tag beads were exchanged in wash buffer (20 mM Tris, 150 mM NaCl, 60 mM imidazole, 2 mM β -mercaptoethanol, pH 7.5) before eluting in elution buffer (20 mM Tris, 150 mM NaCl, 500 mM imidazole, 2 mM β-mercaptoethanol, pH 7.5). For His-tag removal, the eluant was dialyzed into TEV cleavage buffer (20 mM Tris, 150 mM NaCl, 50 mM imidazole, 2 mM β -mercaptoethanol, pH 7.5) overnight at 4 °C in presence of TEV. WT Vt protein and the Vt variants were then collected and run over Ni-NTA agarose beads. The eluent was collected, concentrated and run over a S100 column (GE, Pittsburg, PA) to obtain the highest level of purity using gel filtration buffer A (10 mM Tris, 200 mM KCl, 10 mM imidazole, 2.5 mM MgCl_2_, 1 mM EGTA, 2 mM DTT, pH 7.5) or buffer B (40 mM HEPES, 150 mM NaCl, 2 mM dithiothreitol, pH 7.4). Purified proteins (>96% pure) were concentrated to 100 mM by centrifugation, aliquoted and snap frozen using liquid nitrogen. Protein stocks were then stored at −80 °C.

### Circular Dichroism

To assess the impact of the Vt variants on the conformation and structural stability of Vt, we employed Circular Dichroism (CD) spectroscopy. CD spectra were collected at both near-ultraviolet (350–250 nm) and far-ultraviolet (260–190 nm) spectral regions to monitor secondary and tertiary structural profiles for comparison of WT Vt to the Vt variants using a Jasco J-815 CD spectrophotometer. All spectra were acquired at 20 °C in a buffer containing 10 mM potassium phosphate, 50 mM Na_2_SO_4_, 1 mM dithiothreitol, pH 7.5. Each sample was placed in a 0.1 mm path-length (400 μL) cuvette, and spectra recorded with 0.1 nm data increments at a scanning speed of 50 nm/min. Vt protein concentration of 0.20 mM was used for near-UV and 20 μM for far-UV CD data collection and the resultant spectra averaged over three scans. A set of three independent experiments was performed for near UV, far UV and thermal melt curve respectively.

### Lipid Co-Sedimentation

The following lipids (Avanti Polar Lipids) were employed for lipid cosedimentation assays: (1) 1,2-dioleoyl-*sn*-glycero-3-phosphocholine (PC). (2) 1,2-dioleoyl-*sn*-glycero-3-phosphoethanolamine (PE). (3) 1,2-dioleoyl-*sn*-glycero-3-phospho-L-serine (PS). (4) L-α-phosphatidylinositol-4,5-bisphosphate (PIP_2_; Brain, porcine). The interaction of WT VT and Vt DAFS variants with lipids was assessed by conducting a co-sedimentation assay with unilamellar vesicles (LUVs) as reported previously^25,34^. Comparison of WT Vt and the Vt variants to bind PI and PS was assessed using lipid vesicles containing 60% PE, 40% PC by weight, with either PIP_2_ or PS replacing PE at the concentration indicated. PIP_2_ binding to Vt variants was characterized using vesicles containing 60% PE, 20% PC, and either 20% PS by weight or PIP_2_ replacing PC at the concentration indicated. For example, experiments testing the role of 10% PIP_2_ employed vesicles were composed of 60% PE, 20% PC, 10% PS, and 10% PIP_2_. The desired quantity of lipid was mixed, dried under nitrogen glass and left in a vacuum overnight. Dried lipids were rehydrated in buffer (40 mM HEPES, 150 mM NaCl, 2 mM dithiothreitol, pH 7.4) for 2 hours with constant shaking, and then extruded with 100 nm polycarbonate membranes in a mini-extruder (Avanti) to form large unilamellar vesicles (LUVs) and stored overnight at 4 °C. The following day, LUVs containing 250 μg of lipids and 10 μl of 100 μM Vt protein (in an identical buffer) were added to each vesicle sample, producing a final volume of 100 μl and incubated for 1 hour (at 4°C, under slow constant rotation). The lipids-protein mixture was spun at 120,000 × g in a Beckman TLA100 rotor for 1 hr (4 °C). Supernatants and pellets were separated and run on an SDS-PAGE gel, stained with Coomassie Brilliant Blue, analyzed and quantified with ImageJ Software^74^.

### Actin Co-Sedimentation

To assess actin-binding and actin-bundling (crosslinking) properties of WT Vt and the Vt DAFS variants, we performed actin co-sedimentation assays as reported previously^73^. Briefly, monomeric actin (G-actin) was purified through gel filtration from rabbit muscle acetone powder (obtained from Pel-Freez Biologicals, Rogers, AR) and stored at −80 °C in storage buffer (50 mM imidazole, 100 mM NaCl, 10 mM MgCl_2_, 10 mM EGTA, 0.5 mM DTT, 0.2 mM ATP, pH 7.0). F-actin was prepared by allowing polymerization of G-actin at 100 μM concentration in actin polymerization buffer (10 mM Tris, 200 mM KCl, 10 mM imidazole, 2.5 mM MgCl_2_, 1 mM EGTA, 2 mM DTT, pH 7.5) at room temperature under slow constant rotation for 30 minutes. The heterogeneity of F-actin polymers makes it difficult to quantify F-actin concentrations, so the actin concentrations reported here are based on the G-actin concentration. For the actin binding study, 100 μl samples were prepared by mixing 10 μM Vt variants in actin polymerization buffer and 20 μM F-actin. Samples were then incubated at room temperature for 1 hr and then centrifuged at 185,000 RCF for 60 min. Similarly, to quantify actin bundling/crosslinking, 100 μl samples were prepared containing 10 μM Vt variants and 20 μM F-actin. The samples were incubated at room temperature under slow constant rotation for 1 hr and then centrifuged at 12,000 RCF for 15 min. For both binding and bundling co-sedimentation assays, the supernatant and pellet were separated by centrifugation, resuspended to equal volumes of SDS-PAGE buffer, and analyzed by 15% SDS-PAGE. F-actin binding and bundling was calculated by determining the fraction of Vt protein in the pellets by quantifying the densities of the pellet and supernatant bands. Densitometry was performed using ImageJ^74^.

### Creation of Stable Cell Lines

To generate cell lines that stably express a given Vcn construct, second-generation viral packaging plasmids psPax2 (Addgene Plasmid #12260) and pMD2.G (Addgene Plasmid #12259) were used. To create viral particles, either pRRL-VcnTS (or a variant thereof), along with psPax2 and pMD2.G plasmids, were co-transfected into HEK293-T cells. Three days post-transfection, media containing viral particles was harvested and stored at −80 °C. One day prior to viral transduction, Vcn^−/−^ MEFs were plated in 6-well dishes at a density of 100,000 cells per dish. Cells were transduced with 500 μL of viral mixture in full media supplemented with 2 μg/mL polybrene (Sigma-Aldrich, St. Louis, USA) to enhance viral uptake. After 3 passages, transduced cells were sorted with flow cytometry into several groups based on intensity of the stable construct’s fluorescent signal expression before being frozen down. As previously described, an immunofluorescence-based procedure was used to select Vcn^−/−^ MEFs expressing the Vcn biosensor at levels comparable to endogenous Vcn in unaltered MEFs^38^.

### Cell Culture and Transfection

Vcn^−/−^ MEFs and cell lines expressing VcnTS or VcnTS variants were maintained in high-glucose DMEM with sodium pyruvate (Sigma-Aldrich) supplemented with 10% FBS (Cytiva HyClone, Marlborough, MA), 1% v/v non-essential amino acids (Thermo Fisher Scientific, Waltham, MA), and 1% v/v antibiotic-antimycotic solutions (Sigma-Aldrich). HEK293 cells were maintained in high-glucose DMEM (Sigma-Aldrich) supplemented with 10% FBS (Cytiva HyClone) and 1% v/v antibiotic-antimycotic solution (Sigma-Aldrich). Cells were grown at 37°C in a humidified 5% CO_2_ atmosphere. For transient transfections, Lipofectamine 2000 (Thermo Fisher Scientific) was added according to manufacturer’s instructions.

### Western Blot Analysis

Cells were washed, lysed with lysis buffer [250 mM NaCl, 10% glycerol, 2mM EDTA, 0.5% IGEPAL (Sigma-Aldrich), 50 mM HEPES (Sigma-Aldrich)], and then centrifuged at 13,000 RPM for five minutes. Afterwards, 2x Laemmli sample buffer (Bio-Rad Laboratories, Hercules, California) was added to the lysate for a 1:1 dilution and the sample was denatured at 95 °C for five minutes. Then, the sample was loaded onto a 4–20% gradient Mini-PROTEAN TGX precast gel (Bio-Rad Laboratories) and electrophoresed at 100 V for 70 minutes, before being transferred to a PVDF membrane (Bio-Rad Laboratories) via wet transfer. Membranes were blocked with 5% dry milk in TBST [10 mM Tris-HCl, 100 nM NaCl, 0.1% Tween 20] for 1 hour and then incubated with an anti-GFP primary antibody (Abcam) at a 1:5000 dilution or a GAPDH primary antibody (Santa Cruz, Dallas, TX) at a 1:3000 dilution overnight at 4 °C. Afterwards, the membrane was rinsed three times with TBST and incubated with the appropriate species-specific enzyme-conjugated secondary antibody (Life Technologies), for 1 hour at room temperature. Membranes were later developed using SuperSignal West Pico Chemiluminescent Substrate (Thermo Fisher Scientific). The resulting signal was detected using digital imaging (Bio-Rad Laboratories).

### Cell Seeding for Fluorescence Imaging

For cell imaging, no. 1.5 glass coverslips (Bioptechs, Butler, PA) secured in reusable metal dishes (Bioptechs) were coated overnight at 4°C with 10 μg/mL FN (Fisher Scientific, Pittsburgh, PA) or 0.01% pLL (Millipore Sigma, Burlington, MA). Vcn^−/−^ MEFs expressing a given Vcn construct were then trypsinized, transferred to the prepared glass-bottom dishes at a density of 100,000 cells per dish and allowed to spread for 1 hour on pLL-coated glass and 4 hours on FN-coated glass. For imaging of mature SFs, cells were seeded at 50,000 cells per dish and incubated for 24 hours prior to fixation.

To prepare fixed cells, samples were rinsed quickly with PBS (phosphate-buffered saline) and incubated for 10 min with 4% methanol-free paraformaldehyde (Electron Microscopy Sciences, Hatfield, PA). For live cell imaging, growth media was exchanged for imaging media - Medium 199 (Life Technologies, 11043) supplemented with 10% FBS (Cytiva HyClone), 1% v/v non-essential amino acids (Invitrogen), and 1% v/v antibiotic-antimycotic solution (Sigma Aldrich), at least 1 hour before imaging.

### Immunofluorescence Staining

Fixed cells were subject to permeabilization in 0.1% Triton-X100 (Millipore Sigma, Burlington, MA) for five minutes at room temperature. Cells were then washed with PBS and then blocked in 2% bovine serum albumin (Sigma-Aldrich) diluted in PBS for 30 minutes. To label F-actin, cells were incubated with Alexa-647 labeled phalloidin (Thermo Fisher Scientific) at a 1:100 dilution in 2% BSA for 1 hour. Cells were then washed with PBS three times, preceding imaging.

### FRET and Fluorescence Imaging

An Olympus IX83 inverted epifluorescent microscope (Olympus) equipped with a LambdaLS 300W ozone-free xenon bulb (Sutter Instruments, Novato, CA), sCMOS ORCA-Flash4.0 V2 camera (Hamamatsu, Japan), motorized filter wheels (Sutter Instruments, 10–3), and automated stage (Prior Scientific, H117EIX3) was used to image samples. MetaMorph Advanced software (Olympus, Japan) was used to control the image acquisition. Unless otherwise indicated, all samples were imaged at 60X magnification (Olympus, UPlanSApo 60X/NA1.35 objective, 108nm/pix), with a three-image sensitized emission acquisition sequence. To image mTFP1-Venus sensors, the filter set used includes mTFP1 excitation (Chroma, ET450/30x), mTFP1 emission (Chroma, ET485/20m), Venus excitation (Chroma, ET514/10x), and Venus emission (Semrock, FF01-571/72) filters and a dichroic mirror (Chroma, T450/514rpc). Images were acquired across the acceptor channel (Venus excitation, Venus emission, 1000 ms exposure), FRET channel (mTFP1 excitation, Venus emission, 1500 ms exposure), and donor channel (mTFP1 excitation, mTFP1 emission, 1500 ms exposure).

Alexa 647 phalloidin-labeled cells were imaged in the Cy5 channel of the Semrock DA/FI/TR/Cy-5-4X4 M-C Brightline Sedat filter set with an exposure time of 1000 ms. Images of SFs were acquired in the stain channel (Cy5 excitation, Cy5 emission, 1000 ms exposure).

### Quantitative FRET Efficiency Measurements from Sensitized Emission

For each three-image sensitized emission acquisition, the following process was applied to calculate FRET efficiency. FRET was quantitated on a pixel-by-pixel basis using custom written code in MATLAB (MathWorks, Natick, MA). Prior to FRET calculations, all images were corrected for dark current and uneven illumination, registered, and background subtracted, as described previously^75,76^. FRET imaging of donor-only and acceptor-only samples (cells expressing either a single donor or acceptor fluorescent protein) were used to calculate spectral bleed-through coefficients^76^. Donor bleed-through coefficients (*d*_*bt*_) were calculated for mTFP1 as:

$$d_{bt}=\frac{I_{f}}{I_{d}}$$

where *I*_*f*_ is the FRET-channel intensity and *I*_*d*_ is the donor-channel intensity. Data was binned by donor-channel intensity. Accordingly, acceptor bleed-through coefficients (*a*_*bt*_) were calculated for Venus as:

$$a_{bt}=\frac{I_{f}}{I_{a}}$$

where *I*_*a*_ is the acceptor-channel intensity. In this case, data was binned by acceptor-channel intensity. To correct for spectral bleed-through in experimental data, pixel-by-pixel corrected FRET images (*F*_*c*_) were generated according to the equation:

$$F_{c}=I_{f}-d_{bt}\times I_{d}-a_{bt}\times I_{a}$$

Upon imaging donor–acceptor fusion constructs of constant high or constant low FRET efficiencies^39^, the proportionality constant, *G*, was calculated as:

$$G=-\frac{\Delta \left(\frac{F_{c}}{I_{a}}\right)}{\Delta \left(\frac{I_{d}}{I_{a}}\right)}$$

where Δ indicates the change between two donor-acceptor fusion proteins. Using *G*, FRET efficiency (*E*) was determined:

$$E=\frac{\frac{F_{c}}{G}}{I_{d}\;+\;\frac{F_{c}}{G}}$$

### Cell and FA Segmentation

Individual cells were identified based on closed boundaries drawn by the user on unmasked acceptor channel images. Segmentation of individual FAs was performed using custom-written code in MATLAB implementing the water algorithm and blob analysis^37,75–77^. The results of FA segmentation were output as a mask and applied across all imaging channels, as well as across images resulting from FRET analysis, including FRET efficiency, for visualization of data.

### Quantification of FA Morphometric Properties

For each FA, average FRET efficiency, average acceptor intensity and FA area were calculated as previously described^78^. To calculate FA morphometric parameters such as FA axis ratio and FA orientation, additional blob analysis methods were applied. Central moments were taken of the pixels (*n*) within a FA *F*(*x*, *y*), about the FA centroid (*x*, *>y*), to form an equivalent ellipse^79^. Below, *ij* represents the moment order, where the *ij*^th^ central moment, *u*, is of order *i* + *j*. Therefore, the central moment *u*_*ij*_ is given by the equation:

$$u_{ij}=\sum_{x=0}^{n}\sum_{y=0}^{n}{\left(x-\underline{x}\right)}^{i}{\left(y-\underline{y}\right)}^{j}F(x,y)$$

Using this equation, the second order central moments (*u*_*i*+*j*=2_) were taken and a covariance matrix, *C*_*I(x,y)*_, was subsequently constructed:

$$C_{I(x,y)}=\left[\begin{array}{ll} u_{20} & u_{11}\\ u_{11} & u_{02} \end{array}\right]$$

FA major (*FA*_*maj*_) and minor (*FA*_*min*_) axis lengths were then calculated as a function of *C*_*I*(*x*,*y*)_’s eigenvalues *λ*_1_, *λ*_2_ respectively:

$$FA_{maj}=\sqrt{\lambda_{1}}=\sqrt{2\left(\left(u_{20}+u_{02}\right)+\sqrt{{\left(u_{20}-u_{02}\right)}^{2}+4{\left(u_{11}\right)}^{2}}\right)}$$

$$FA_{min}=\sqrt{\lambda_{2}}=\sqrt{2\left(\left(u_{20}+u_{02}\right)-\sqrt{{\left(u_{20}-u_{02}\right)}^{2}+4{\left(u_{11}\right)}^{2}}\right)}$$

Consequently, FA axis ratio (*FA*_*axrat*_) was determined as:

$$FA_{axrat\;}=\frac{FA_{maj}}{FA_{min}}$$

Lastly, FA orientation from the x-axis was calculated as:

$$\theta_{FA}=(\frac{2\left(u_{11}\right)}{u_{20}\;-\;u_{02}\;+\;\sqrt{{\left(u_{02}\;-\;u_{20}\right)}^{2}\;+\;4{\left(u_{11}\right)}^{2}}})$$

where *u*_20_ > *u*_02_ and *θ*_*FA*_ is in radians, or

$$\theta_{FA}=\left(\frac{u_{02}\;-\;u_{20}\;+\;\sqrt{{\left(u_{02}\;-\;u_{20}\right)}^{2}\;+\;4{\left(u_{11}\right)}^{2}}}{2\left(u_{11}\right)}\right)$$

where *u*_02_ > *u*_20_ and *θ*_*FA*_ is in radians.

### Cytosol Segmentation

To isolate signals from the cytosol, a cytosol mask was generated by dilating the FA mask by a scale factor of 10 and then inverting the newly generated mask within cell boundaries. Cytosol masks were then applied to acceptor and FRET images of cells with cytosolic signal above background pixel intensity.

### Quantification of SF Data

Custom written code in MATLAB was used to identify and analyze actin SFs. Briefly, the structure tensor *S*_0_ of each SF pixel was computed to determine maximal gradients of actin intensity and the orientation of SFs on a pixel-by-pixel basis^80^.

Image I(*p*), where each pixel *p*, at position (*x, y*) has the structure tensor:

$$S_{0}(p)=\left[\begin{array}{cc} {\left(I_{x}(p)\right)}^{2} & I_{x}(p)I_{y}(p)\\ I_{x}(p)I_{y}(p) & {\left(I_{y}(p)\right)}^{2} \end{array}\right]$$

The eigenvectors, **e**_**1**_ and **e**_**2**,_ and eigenvalues, **l**_**1**_ and **l**_**2,**_ of each pixel’s structure tensor were determined. For each pixel, the angle formed by the components of the larger eigenvector (**e**_**1**_**)** was calculated in radians as the SF orientation (*θ*_*SF*_):

$$\theta_{SF}=\left(\frac{e_{1y}}{e_{1x}}\right)$$

Images of *θ*_*SF*_ and **l**_**1**_ (magnitude of the actin intensity gradient) were consequently generated. SF masks were generated by isolating pixels of the **l**_**1**_ image above a desired actin intensity magnitude^81^. SF masks were applied to the *θ*_*SF*_ image to isolate orientations of individual SFs. The standard deviation of SF orientations per cell was calculated to assess the uniformity, or lack thereof, of SF orientations in the cell.

Force generated via SFs is known to affect local focal adhesion initiation and maturation^82^. Thus, we sought to characterize SF characteristics corresponding to nearby FAs. A Voronoi tessellation can be used to partition a plane into smaller regions by using a set of points within the plane to nucleate smaller regions^83^. The Voronoi tessellation operation was used on FA centroids obtained from blob analysis to create regions of local SFs within the cell boundary of actin-labeled, VcnTS or VcnTS E1015A E1021A expressing Vcn^−/−^ MEFs. Within each Voronoi region, local SF density (measured as the percent area of SFs in a Voronoi region) was quantified. For each cell, the local SF densities of all the Voronoi regions were averaged. To obtain a representative SF orientation per local region, each cell’s Voronoi tessellation mask was applied to *θ*_*SF*_ images and a single vector corresponding to SF orientation per region was calculated. From blob analysis, the orientation of each Voronoi region’s respective focal adhesion was obtained. Relative orientation (*θ*_*Rel*_) of a local averaged SF orientation to its respective FA orientation was calculated:

$$\theta_{Rel}=\left|\theta_{SF}-\theta_{FA}\right|$$

*θ*_*Rel*_ for each local region was then averaged per cell.

### Directed Cell Migration Assay

Transwell inserts with 8-μm pores (Corning, Corning, NY) were prepared with fibronectin, as previously described^38^. Briefly, 10 μg/mL of fibronectin solution was used to coat the underside of the transwell insert, and the insert was then placed in serum-free media. Cells were serum-starved for 2 hours prior to seeding. For the seeding procedure, ~16,000 cells in serum-free media were plated into the upper chambers of each well. Cells were allowed to migrate for 4 hours at 37˚C. After migration, cells were fixed in 4% methanol-free paraformaldehyde and permeabilized in 0.1% Triton X100 solution. Using a cotton swab, non-migratory cells from the upper chamber were gently removed. The remaining cells on the underside were stained with Hoechst (Thermo Fisher Scientific) to visualize nuclei. The chambers were subsequently washed with 1x PBS. The chambers were then imaged at 10x on the previously mentioned epi-fluorescence microscope. To analyze the images, rolling ball background subtraction was performed in ImageJ (NIH), and cell nuclei were counted per image frame via the Analyze Particles tool.

### Statistical Analysis

All experimental data sets were initially compared using a Welch’s test for unequal variances. An unpaired 2-tailed t-test was performed when comparing two means. One-way ANOVA followed by post-hoc Tukey (equal variances) or post-hoc Steel-Dwass test (unequal variances) was performed for multiple comparisons using Student Version of Origin software (OriginLab Corporation, MA-01060, USA) or JMP Pro (SAS Institute, NC-27519, USA). All data were presented as mean ± S.E.M. unless otherwise noted. The values for *N*, *p*, and statistical test performed are listed in the figure captions. Tables with *p*-values for construct comparisons are presented in the supplemental document.

## Data Availability

The data that support the findings of this study are available from corresponding authors upon reasonable request.
